# Cancer and Relationship Dissolution: Perspective of Partners of Cancer Patients

**DOI:** 10.3389/fpsyg.2021.624902

**Published:** 2021-05-21

**Authors:** Bahar Nalbant, André Karger, Tanja Zimmermann

**Affiliations:** ^1^Department of Psychosomatics and Psychotherapy, Hannover Medical School, Hanover, Germany; ^2^Medical Faculty, Heinrich-Heine University, Institute for Psychosomatic Medicine and Psychotherapy, University Hospital, Düsseldorf, Germany

**Keywords:** cancer, survivorship, relationship satisfaction, depression, anxiety, relationship dissolution, partners

## Abstract

**Purpose:**

Cancer can be a burden on the relationship and even lead to relationship dissolution. Previous studies about the impact of cancer on close relationships almost exclusively involve cancer patients. So far, little is known about the views of spouses. Therefore, this study focuses on partners or ex-partners of cancer patients.

**Methods:**

In this cross-sectional study, *N* = 265 partners or ex-partners of cancer patients are examined regarding a possible separation, the reasons for separation and the influence of the cancer on the relationship. In addition, predictors of separation and the positive or negative perception of the impact of cancer on the relationship were investigated.

**Results:**

The separation rate (23.4%) was marginally lower than in the general population in Germany (35.79%). The most frequent reason for separation was the death of the cancer patient (59.6%), followed by relationship problems (26.9%), and the cancer disease itself (9.6%). Among those who were separated, 57.4% reported that cancer contributed to the separation. On average, the influence of cancer on relationship dissolution is indicated with 82.9%. Also, for those who stayed together, 83.7% reported an impact of the cancer on the relationship, of which 55.9% reported a negative impact. Logistic regressions indicated that higher levels of depression were associated with greater odds of a more negative perception of the influence of cancer on the relationship, whereas a more satisfied relationship tended to be associated with a more positive perception. Those who had no psychological treatment in the past, lower anxiety levels and lower relationship satisfaction had an increased risk of separation. Overall, relationship satisfaction was significantly lower than in the general population in Germany.

**Conclusion:**

In particular, psychological factors such as depression and anxiety as well as relationship satisfaction appear to be factors influencing separation and the perception of the influence of cancer on the relationship as positive or negative. Therefore, it seems to be reasonable to consider these aspects in the psychosocial support and also to include the partners in order to achieve a stable and satisfied relationship which has a positive effect on health and psychological well-being.

## Introduction

Cancer and its therapy can present challenges and burdens that can last over time, not only for patients but also for their intimate partners and the relationship (Kayser and Scott, 2008; Aizer et al., 2013). Around 50% of cancer patients show high levels of distress (Mehnert et al., 2018), including clinically significant emotional distress and/or unrecognized or untreated psychosocial conditions as a result of cancer (Grassi, 2020). For the majority of patients, partners are the primary source of support (Manne and Badr, 2008; Forsythe et al., 2014). However, partners experience comparable levels of psychological distress, depression, and anxiety, as well as reduced quality of life (Sjovall et al., 2009; Sklenarova et al., 2015; Zimmermann, 2015; Brandao et al., 2017), and high distress and low relationship satisfaction (Li and Loke, 2013). Therefore, the support of the partner can also be influenced by their own stress and this can have a stressful effect on the relationship.

Marital adjustment is important for the health and psychological well-being of both partners. Higher marital quality is associated with better health (Robles et al., 2014). In the specific context of cancer, the relationship has a positive impact on timing of diagnosis, treatment outcome, and cancer mortality. Having a partner is beneficial after cancer (Dasgupta et al., 2016). For example, Buja et al. (2018) found in their systematic review that unmarried patients have a higher risk of advanced cancer or melanoma at the time of diagnosis. In addition, unmarried patients have a higher risk of metastatic cancer, undertreatment, as well as death due to cancer than married patients (Aizer et al., 2013). Cancer survivors who receive more social support from their partners are more likely to successfully cope with the challenges of a cancer diagnosis, including managing depression and anxiety, maintaining a healthy lifestyle and positive attitude, and coping with occupational and financial problems (Kvikstad et al., 1995). In general, most individuals in close relationships find ways to cope and adapt to the challenging stressors of cancer. However, when dyadic adjustment to cancer-related distress fails, the relationship breaks down (Foster et al., 2009; Kirchhoff et al., 2012). A subset of patients and their partners are at higher risk for separation and divorce (Carlsen et al., 2007; Karraker and Latham, 2015; Sbarra et al., 2015) compared with the general population.

Some studies show that marital stress associated with cancer may lead to an increased risk of separation and divorce compared with the general population (Karraker and Latham, 2015); others found that the risk of divorce is no greater in cancer survivors than in the general population (Carlsen et al., 2007). Studies have also found gender differences, with female patients being significantly more likely to divorce than male patients (Syse, 2008; Glantz et al., 2009; Karraker and Latham, 2015). Nevertheless, the results are inconsistent. A meta-analysis showed that couple-based interventions had a small to medium impact on cancer patients’ physical health. Partners were able to derive moderate effects from couple-based interventions on improving sexual relations (Li et al., 2020). Another systematic review for psychological interventions targeting partners of cancer patients showed positive effects related to social support, distress, and communication for partners and patients (Kleine et al., 2019). A study examining the processes of intimacy and psychological distress in couples with different cancers shows an improvement in relationship intimacy through disclosure of cancer-related concerns. This could make it easier for both partners to adjust to the disease (Manne et al., 2010). In the context of these findings, the involvement of partners of cancer patients is a crucial criterion for a stable relationship and good coping with the disease.

Most studies addressing cancer and relationship dissolution examine patients. To the best of our knowledge, there have been few studies that bring in the perspective of the partners on the impact of cancer on relationship quality and continuation (Sjovall et al., 2009; Drabe et al., 2013). Demands for future studies to also survey partners or ex-partners should be addressed here (Stephens et al., 2016). Stephens et al. (2016) stated that including ex-spouses may help “to understand relationships among cancer-related problems and relationship dissolution” (p. 872). Specifically, the inclusion of ex-partners may help to more accurately capture reasons for separation. Ex-partners were defined as individuals who had been in a relationship with a cancer patient but had ended it. In addition to the patient’s perspective, it seems useful to capture the partners’ perspective as well. Because most previous studies have focused on cancer patients and separation, examing partners could provide further insight into the impact of cancer on relationships and, in particular, on separation. The research questions of the present study focus on the influence of partners’ sociodemographic factors such as age, gender, separation of own parents, children, or medical factors (own disease, psychological treatment) and psychological factors such as depression, anxiety, distress, quality of life, and relationship satisfaction on marital stability, as well as the influence of cancer on their relationship and influencing factors from the partners’ perspective. Therefore, the present study focuses on relationship dissolution among partners of cancer patients and examines (1) the frequency of relationship dissolution and the reasons for relationship dissolution. In addition, (2) the impact of cancer on the relationship, and (3) the predictors of relationship dissolution are analyzed.

## Materials and Methods

### Participants

Partners or ex-partners (*N* = 265) of cancer patients participated in the study. Demographic and psychological factors as well as differences between partners and ex-partners are shown in Table 1. The mean duration of those currently in a relationship with the cancer patient (*n* = 203) was 20.46 (SD = 13.59) years. The mean relationship duration until cancer diagnosis was 17.98 (SD = 12.86) years. For those who separated from the cancer patient (*n* = 62), the mean relationship duration to separation was 19.10 (SD = 14.65) years. The most frequent types of cancer among patients were colon cancer (18.5%), lung cancer (12.8%), and breast cancer (10.6%). In 55.8% of patients, the cancer was a primary disease, in 3.4% a secondary disease and in 17% a recurrence. A total of 21.1% reported that the cancer was currently cured. 57.4% of patients were currently receiving medical treatment. Of the medical treatments, 19.1% received surgery, 48.7% received chemotherapy, 19.1% received radiation.

**TABLE 1:** Demographics, relationship-related and health-related variables of the total sample (*N* = 265) as well as for partners who are in a relationship with the cancer patient at the time of the study (*n* = 203) and those who have separated (*n* = 62).

**Variable**	***N* = 265 total sample**	***N* = 203 in relationship with the cancer patient**	***N* = 62 separated from the cancer patient**	**Differences**
*Demographics*				
Sex, *n* (%)				n.s.
Female	197 (74.3)	152 (77.2)	45 (22.8)	
Male	68 (25.7)	51 (75.0)	17 (25.0)	
Mean age (SD)	50.32 (12.58)	50.43 (12.36)	49.97 (13.36)	n.s.
Education, *n* (%)^1^				n.s.
Less than 10 years	42 (15.9)	31 (73.8)	11 (26.2)	
10 years	80 (30.2)	66 (82.5)	14 (17.5)	
More than 10 years	140 (52.8)	105 (75.0)	35 (25.0)	
Job status, *n* (%)				n.s.
Full-time employed	114 (43.0)	89 (78.1)	25 (21.9)	
Half-time employed	63 (23.8)	51 (81.0)	12 (19.0)	
Retired	51 (19.2)	39 (76.5)	12 (23.5)	
In sick leave	15 (5.7)	9 (60.0)	6 (40.0)	
Homework	11 (4.2)	10 (90.9)	1 (9.1)	
Study/training	6 (2.3)	2 (33.3)	4 (66.7)	
Unemployed	5 (1.9)	3 (60.0)	2 (40.0)	
Children				n.s.
No	80 (30.2)	60 (75.0)	20 (25.0)	
Yes	185 (69.8)	143 (77.3)	42 (22.7)	
Separation of own parents, *n* (%)				n.s.
No	62 (23.4)	44 (71.0)	18 (29.0)	
Yes	203 (76.6)	159 (78.3)	44 (21.7)	
*Medical variables*				
Own somatic illness, *n* (%)				n.s.
Yes	107 (40.4)	80 (74.8)	27 (25.2)	
No	158 (59.6)	123 (77.8)	35 (22.2)	
Psychological/psychiatric treatment in the past, *n* (%)				*X*^2^ = 12.80, df = 1, *p* < 0.001
Yes	91 (34.3)	58 (63.7)	33 (36.3)	
No	174 (65.7)	145 (83.3)	29 (16.7)	
Current psychological/psychiatric treatment, *n* (%)				n.s.
Yes	42 (15.8)	30 (71.4)	12 (28.6)	
No	223 (84.2)	173 (77.6)	50 (22.4)	
Cancer diagnosis of the cancer patient, *n* (%)^2^				n.s.
Colon cancer	49 (18.5)	37 (75.5)	12 (24.5)	
Lung cancer	34 (12.8)	28 (82.4)	6 (17.6)	
Breast cancer	28 (10.6)	21 (75.0)	7 (25.0)	
Urological cancer	27 (10.2)	18 (66.7)	9 (33.3)	
Stomach cancer	22 (8.3)	13 (59.1)	9 (40.9)	
Hematological cancer	22 (8.3)	20 (90.9)	2 (9.1)	
Prostate cancer	18 (6.8)	15 (83.3)	3 (16.7)	
Mean time since diagnosis in months (SD, range)	44.2 (55.9, 0–310)	37.7 (52.2)	65.3 (62.5)	*t*(261) = 3.47, *p* = 0.001
Current disease status of the cancer patient, *n* (%)				*X*^2^ = 24.51, df = 4, *p* < 0.001
Primary disease	148 (55.8)	118 (79.7)	30 (20.3)	
Cancer in remission	56 (21.1)	43 (76.8)	13 (23.2)	
Cancer recurrence	45 (17.0)	36 (80.0)	9 (20.0)	
Secondary disease	9 (3.4)	6 (66.7)	3 (33.3)	
Not known	7 (2.6)	0 (0)	7 (100)	
Current treatment status of the cancer patient, *n* (%)				*X*^2^ = 40.1, df = 2, *p* < 0.001
Treatment ongoing	152 (57.4)	137 (90.1)	15 (9.9)	
Treatment completed	106 (40.0)	64 (60.4)	42 (39.6)	
Not known	7 (2.6)	2 (28.6)	5 (71.4)	
*Psychological variables M (SD)*				
Distress (DT)	6.4 (2.5)	6.4 (2.4)	6.3 (2.7)	n.s.
Depression (PHQ-9)	9.5 (5.9)	8.9 (5.8)	11.1 (6.2)	*t*(263) = 2.60, *p* = 0.01
Anxiety (GAD-7)	8.1 (5.4)	8.0 (5.3)	8.4 (5.7)	n.s.
Health-related quality of life (EQ-5D)	83.5 (14.8)	84.0 (14.9)	81.8 (14.4)	n.s.
State of health (EQ VAS)	69.2 (24.4)	70.0 (24.5)	66.7 (24.3)	n.s.
Relationship satisfaction (QMI)	35.2 (9.0)	35.5 (9.1)	34.4 (8.6)	n.s.

*n.s. = not significant.*

*^1^*n* = 3 (1.1%) others.*

*^2^cancers below 5% (gynecological cancer 3.8%, head and neck cancers 4.5%, melanoma 4.5%, brain tumor 2.3%, and others 6.8%).*

*QMI-D, Quality of Marriage Index; DT, NCCN Distress Thermometer; PHQ-9, Patient Health Questionnaire; GAD-7, Generalized Anxiety Disorder Seven Item Scale; EQ-5D, EuroQol Five Dimensions Questionnaire; and EQ VAS, EQ-5D Visual Analog Scale (0–100). The percentages refer to the respective rows.*

The mean distress score was 6.4 (SD = 2.5). In addition, 78.5% (*n* = 208) of participants reported an elevated distress level. The mean severity of depressive symptoms was mild to moderate with *M* = 9.5 (SD = 5.9). Minimal depression scores were found in 23.4% (*n* = 62), mild in 34.7% (*n* = 92), moderate in 22.3% (*n* = 59), moderately severe in 12.5% (*n* = 33), and severe in 7.2% (*n* = 19) of the partners. The mean score of anxiety (*M* = 8.1, SD = 5.4) was mild. 29.8% of participants showed minimal anxiety scores, 35.5% mild, 20.4% moderate and 14.3% severe. The health-related quality of life-visual analog scale (*M* = 69.2, SD = 24.4) was below the population mean [*M* = 77.1, SD = 17.8; *t*(2285) = 6.47, and *p* < 0.001] as well as the EQ-5D sum score (*M* = 83.5, SD = 14.8) compared to the German general population [*M* = 91.7, SD = 13.1; *t*(2285) = 9.43, and *p* < 0.001] (Hinz et al., 2006). Relationship satisfaction (*M* = 35.2, SD = 9.0) was lower than in the general population in Germany [*M* = 38.65, SD = 6.91; *t*(1694) = 7.09, and *p* < 0.001]. In addition, 32.1% (*n* = 85) of the sample was below the cut-off of 34, which indicates an unsatisfied relationship (Zimmermann et al., 2019). Associations between time since cancer diagnosis were shown only for distress (*r* = −0.21, *p* = 0.001), not for depression, anxiety, quality of life, or relationship satisfaction. No differences were found for depression [*X*^2^(16) = 25.6, *p* = 0.06] or distress [*X*^2^(4) = 7.7, *p* = 0.10] and current disease status, but differences emerged for anxiety and disease status [*X*^2^(12) = 30.5, *p* = 0.002] with higher anxiety when cancer recurred or it was a first disease. No differences were found in current treatment status (treatment ongoing vs. treatment completed) related to depression [*X*^2^(8) = 4.9, *p* = 0.77] or anxiety [*X*^2^(6) = 9.2, *p* = 0.16], but there were differences in distress [*X*^2^(2) = 6.9, *p* = 0.03]. When the patient was under current medical treatment, no 128 (48.3%) of the partners experienced distress above the cut-off, whereas 28.3% (*n* = 75) were above the cut-off when treatment was completed. In sum, the present sample appeared to have increased psychological distress, mild to moderate depression, mild anxiety, and lower health-related quality of life. Satisfaction with the relationship was also lower than in a German comparison sample.

### Procedure

For the analysis of separation and divorce, the study population was restricted to those partners who were either living with the cancer patient or married at the time of the cancer diagnosis. An online questionnaire (created with Questback EFS Survey), accessible via an URL, was used to collect data. The survey period ran from November 1st, 2017 to September 1st, 2020. The participants were asked about their mental and physical health, the quality of their relationship, and the separation events during and after their partners’ cancer. Participation in this nationwide online survey was voluntary, anonymous, and free of charge. Inclusion criteria were: age 18 years and older, presence of a partner’s cancer diagnosis currently or in the past, and the absence of severe mental impairments. Several cancer-related or oncological organizations (Cancer Society of Lower Saxony, Cancer Society of North Rhine-Westphalia, Network of Comprehensive Cancer Centers/German Cancer Aid) supported recruitment. Participants were informed about the study via mail, flyers or postal cards by oncological centers of hospitals, advice centers, or support groups. In addition, the study was advertised on the websites of Hannover Medical School and the University Hospital Düsseldorf Germany.

Ethical approval for this study was obtained from the ethics committees of the Hannover Medical School (number 3653-2017) and the University Hospital Düsseldorf (number 2017114500).

### Measures

#### Depression

The German version of the depression scale of the Patient Health Questionnaire (PHQ-9; Kroenke et al., 2001) was used to measure the severity of depression symptoms. It consists of nine items answered on a 4-point scale (0 = not at all to 3 = almost every day), e.g., “Over the last 2 weeks, how often have you been bothered by any of the following problems? Little interest or pleasure in doing things.” The sum of item scores includes a range from 0 to 27. From a value of 10, the diagnosis of depression is proposed (10–14 = mild, 15–19 = moderate, and 20–27 = severe). In the original sample the PHQ-9 was a reliable (α = 0.89) and valid measure of depression severity (Kroenke et al., 2001). Cronbach’s alpha in the present study was α = 0.88.

#### Anxiety

The German version of the self-report questionnaire of the Generalized Anxiety Disorder Seven Item Scale (GAD-7; Spitzer et al., 2006) was used to measure the severity of anxiety symptoms. The questionnaire includes seven items ranged from 0 = not at all, 1 = several days, 2 = more than half of the days, to 3 = nearly every day, e.g., “Over the last 2 weeks, how often have you been bothered by the following problems? Feeling nervous, anxious or on edge.” The items were added (range from 0 to 21). Higher values indicating higher severity of generalized anxiety symptoms. Scores of 5, 10, and 15 represent cut-off points for mild, moderate, and severe anxiety, respectively. Cronbach’s alpha in the present study was α = 0.91.

#### Health-Related Quality of Life

The German version of the EuroQol Five Dimensions Questionnaire (EQ-5D; Hinz et al., 2006) was used to measure the health-related quality of life with five items (mobility, self-care, usual activities, pain/discomfort, and anxiety/depression; e.g., Mobility: I have no problems in walking about; I have some problems in walking about; and I am confined to bed) answered on a three-point scale (1 = no problems, 2 = moderate problems, and 3 = extreme problems). The score was calculated as the sum of item scores minus 5, multiplied with 10, and subtracted from 100 (Hinz et al., 2006). This calculation resulted in a range from 0 to 100. Higher values indicating higher quality of life. Cronbach’s alpha in the present study was α = 0.61. Furthermore, the EQ-5D Visual Analogue Scale (EQ-VAS) measures the state of health on a horizontal slider bar ranging from 0 = the worst imaginable state of health to 100 = the best imaginable state of health. Higher values indicating better state of health.

#### Psychosocial Distress

The German version of the NCCN Distress Thermometer (DT; Mehnert et al., 2006) was used to measure psychosocial distress. The DT is a screening tool that has been used in psycho-oncologic research worldwide in order to detect clinically significant levels of distress in patients with cancer (Donovan et al., 2014). The DT consists of a single item which assesses the global level of distress that has been experienced in the past week, including the present day [“Please circle the number (0–10) that best describes how much distress you have been experiencing in the past week including today”]. The scale ranges from 0 (no distress) to 10 (extreme distress) with a cut-off score of 5 indicating a clinically significant level of distress. The DT has been validated in cancer patients with different diagnoses and disease stages (Donovan et al., 2014).

*Relationship quality* was measured with the German version of the Quality of Marriage Index (QMI-D; Zimmermann et al., 2015, 2019). Five of the six items are answered on a seven-point Likert scale (1 = very strong rejection to 7 = very strong agreement; e.g., “Our marriage is strong”). A global item is rated on a 10-point scale (1 = very unhappy to 10 = perfectly happy). The total value ranges between 6 and 45, lower values stand for a lower relationship quality. A cut-off value of 34 is given. Values above the cut-off indicate a satisfied relationship. Reliability in the original sample was high with α = 0.94 (Zimmermann et al., 2019). Cronbach’s alpha in the present study was α = 0.95.

In addition, we asked partners whether cancer had affected their relationship (yes vs. no) and, if so, how (positively or negatively). For those who separated, the survey asked “Do you think cancer was a contributing factor to the separation?” and asked to provide a percentage if the answer was yes.

### Statistical Analyses

For descriptive statistics, percentages, frequencies, mean values and standard deviations were calculated. In order to determine differences between groups, *t*-tests for independent samples and chi^2^-tests were computed. Categorical dependent variables (relationship dissolution, positive vs. negative perception of the influence of cancer on relationship) were predicted using logistic regression. In logistic regression model, age, gender, children, parental separation, physical disease, psychological treatment in the past, quality of life (EQ-5D), depression (PHQ-9), anxiety (GAD-7), psychological distress (DT), and relationship satisfaction (QMI) were used as independent variables. For all predictors, tolerance was above 0.25 indicating that no severe multicollinearity was present. For every regression model, graphical residual analysis indicated that no severe heteroscedasticity was present either. Results of the logistic regression models were reported as odds ratios (ORs) with 95% confidence intervals (CIs). *P*-values less than 0.05 were considered statistically significant. All statistical analyses were executed with SPSS 26.0.

## Results

### Relationship Dissolution: Frequency and Reasons

**FIGURE 1:**
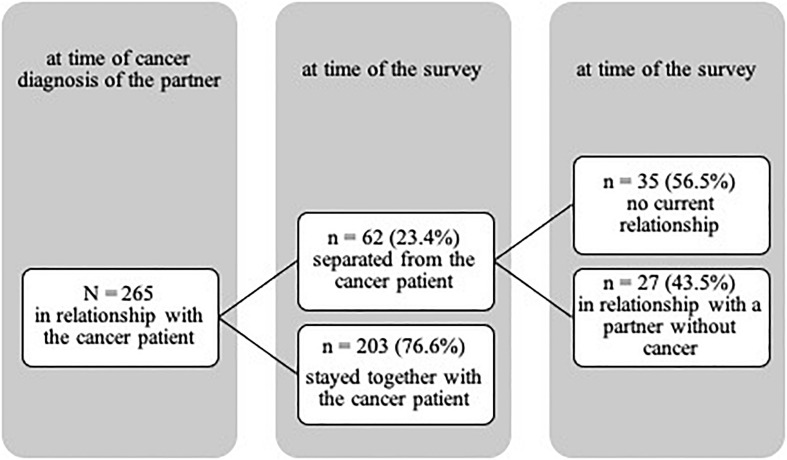
Changes in relationship status of the sample between the time of the cancer diagnosis of the partner and the time of the survey.

The separation rate of the participants (23.4%, *n* = 62) was lower than in the general population in Germany (35.79%; chi^2^ = 3.59, and *p* = 0.058; Statistisches Bundesamt, 2020). Of those who had separated from the cancer patient (*n* = 62), *n* = 27 were in a new relationship with a non-cancer partner at the time of the survey. *N* = 35 participants were not currently in a relationship (see Figure 1). The most frequent reason for separation was the death of the cancer patient (59.6%, *n* = 31), followed by relationship problems (26.9%, *n* = 14), and the cancer itself (9.6%, *n* = 5). However, if those participants are excluded for whom the reason for separation was “death of the patient” (*n* = 31), the separation rate was 13.2%, significantly lower than the separation rate in Germany.

### Influence of Cancer on Relationship

Of those who were separated from the cancer patient (*n* = 62), 57.4% (*n* = 35) indicated that cancer contributed to the separation. On average, the impact of cancer on relationship dissolution was reported as *M* = 82.9% (SD = 25.03). Of those who were currently in a new relationship (*n* = 27), 59.3% (*n* = 16) reported that their former partner’s cancer also had an impact on their current relationship, with the majority (69.2%) reporting a positive impact. Of those who remained with the cancer patient, 83.7% (*n* = 170) reported that cancer had influenced their relationship; 44.1% (*n* = 75) considered this influence to be positive, 55.9% (*n* = 95) considered it to be negative.

A logistic regression analysis was performed for those *n* = 170 who remained with the cancer patient and perceived an influence of cancer on the relationship, with the type of influence (positive or negative) of cancer on the relationship as the dependent variable and age, gender, children, parental separation, physical disease, psychological treatment in the past, EQ-5D, PHQ-9, GAD-7, DT, and QMI as predictor variables (see Table 2). A total of 170 cases were analyzed and the full model predicted type of influence (omnibus chi-square = 35.39, df = 12, and *p* < 0.001). The model accounted for between 18.8 and 25.2% of the variance in type of influence, with overall 70% of accurate predictions. Table 2 gives coefficients and the Wald statistic and associated degrees of freedom and probability values for each of the predictor variables. This shows that only depression and relationship satisfaction reliably predicted the type of influence of cancer on the relationship. The values of the coefficients showed that higher depression and lower relationship satisfaction were associated with negative influence of cancer on the relationship by a factor of 1.12 and 0.91, respectively (depression: 95% CI 1.01–1.24, relationship satisfaction: 95% CI 0.87–0.95).

**TABLE 2:** Coefficients from binary logistic regression of negative or positive influence of cancer on relationship (*N* = 170).

**Predictor**	**β**	***SE*_β_**	***OR***	**Wald χ^2^**	***p***
Age	–0.02	0.02	1.00	0.02	0.90
Gender	–0.01	0.46	1.00	0.00	0.99
Number of children	–0.54	0.38	0.58	1.98	0.16
Parental separation	–0.63	0.44	0.53	2.06	0.15
Own physical disease	–0.30	0.41	0.74	0.55	0.46
Psychological treatment in the past	–0.07	0.39	0.93	0.03	0.86
Health-related quality of life (EQ-5D)	–0.02	0.02	0.99	0.77	0.38
State of health (EQ VAS)	–0.00	0.01	1.00	0.22	0.64
Depression (PHQ-9)	0.11	0.05	1.12	4.33	**0.04**
Anxiety (GAD-7)	–0.02	0.06	0.98	0.14	0.71
Distress (DT)	0.01	0.10	1.01	0.01	0.92
Relationship satisfaction (QMI)^a^	–0.10	0.02	0.91	16.46	**0.00**

*Influence of cancer on relationship was coded as 1 = positive and 2 = negative. DT, NCCN Distress Thermometer; PHQ-9, Patient Health Questionnaire; GAD-7, Generalized Anxiety Disorder Seven Item Scale; EQ-5D, EuroQol Five Dimensions Questionnaire; EQ VAS, EQ-5D Visual Analog Scale; and OR, odds ratio. Significant coefficients in bold.*

*^*a*^Relationship satisfaction with the cancer patient.*

### Predictors of Relationship Dissolution

Differences between those who separated and those who stayed with the cancer patient were found for psychological treatment in the past and depression, with those who separated showing higher scores (*p* = 0.01; see Table 3).

**TABLE 3:** Coefficients from binary logistic regression of relationship dissolution (*N* = 233)^1^.

**Predictor**	**β**	***SE*_β_**	***OR***	**Wald χ^2^**	***p***
Age	0.02	0.02	1.02	0.84	0.34
Gender	0.71	0.49	2.03	2.06	0.15
Number of Children	–0.21	0.47	0.81	0.20	0.66
Parental separation	0.20	0.52	1.23	0.15	0.70
Own physical disease	–0.45	0.51	0.64	0.80	0.37
Psychological treatment in the past	–1.24	0.44	0.29	8.09	**0.004**
Health-related quality of life (EQ-5D)	–0.01	0.02	0.99	0.54	0.46
State of health (EQ VAS)	0.001	0.01	1.00	0.002	0.96
Depression (PHQ-9)	–0.12	0.07	0.89	3.03	0.08
Anxiety (GAD-7)	0.20	0.08	1.22	5.88	**0.02**
Distress (DT)	0.01	0.11	1.01	0.01	0.95
Relationship satisfaction (QMI)^a^	0.04	0.02	1.04	3.93	**0.05**

*^1^*n* = 31 were excluded due to death of the patient as separation reason. Relationship dissolution was coded as 1 = yes (partnership with the cancer patient did not exist at the time of the survey) and 2 = no (partnership still exist).*

*DT, NCCN Distress Thermometer; PHQ-9, Patient Health Questionnaire; GAD-7, Generalized Anxiety Disorder Seven Item Scale; EQ-5D, EuroQol Five Dimensions Questionnaire; EQ VAS, EQ-5D Visual Analog Scale; and OR, odds ratio. Significant coefficients in bold.*

*^*a*^Relationship satisfaction with the cancer patient.*

To analyze factors influencing relationship dissolution, those who stayed with the cancer patient and those who separated were compared. However, those for whom the death of the patient was the reason for separation (*n* = 31) were excluded. In this case it can be assumed that the relationship would continue without the death of the patient. A logistic regression analysis was performed with relationship dissolution as the dependent variable, and age, gender, children, parental separation, physical disease, psychological treatment in the past, EQ-5D, PHQ-9, GAD-7, DT, and QMI as predictor variables. A total of 233 cases were analyzed and the full model significantly predicted relationship dissolution (omnibus chi-square = 26.74, df = 12, and *p* = 0.008). The model accounted for between 10.8 and 20.2% of the variance in relationship dissolution status, with overall 87.6% of accurate predictions. Table 3 gives coefficients and the Wald statistic and associated degrees of freedom and probability values for each of the predictor variables. This shows that only psychological treatment in the past, anxiety and relationship satisfaction reliably predicted relationship dissolution. The values of the coefficients revealed that no psychological treatment in the past, lower anxiety scores and lower relationship satisfaction are associated with an increase in the odds of relationship dissolution by a factor of 0.29, 1.22, and 1.04, respectively (psychological treatment in the past: 95% CI.12 − 0.68, anxiety: 95% CI 1.04 – 1.43, and relationship satisfaction: 95% CI 1.00 – 1.09).

## Discussion

The aim of the present study was to investigate partnership dissolution in the context of cancer among partners or ex-partners of cancer patients. For this purpose, the frequency of dissolution and its reasons as well as the influence of cancer on the relationship and predictors of dissolution from the partners’ perspective were to be examined. Additionally, the subjective psychological and physical status of partners of cancer patients was included in the evaluation.

The dissolution rate among partners was lower than the separation rate in the general German population. This is consistent with other studies showing that cancer survivors were not at higher risk of divorce than the general population (Carlsen et al., 2007; Laitala et al., 2015; Stephens et al., 2016). Death of the cancer patient was the most frequent reason for relationship dissolution. However, half of the separated partners also gave other reasons (e.g., relationship problems) for separation. Relationship problems appear to be a key contributor to separation.

More than half of those who were separated stated that cancer contributed to elationship dissolution. On average, the influence of cancer on relationship dissolution was as high as 82.9%. Even among those who had separated from the cancer patient and were in a new relationship, the cancer also had an impact on the new relationship – but for the majority, a positive one. It is possible that experiencing the cancer in the partner has also changed their own attitudes. Research on post-traumatic growth after cancer shows comparable positive effects in patients and their partners (Zwahlen et al., 2010). Thus, it is possible that these positive effects (such as a sense of togetherness, shared strength, and being able to rely on each other) can also be transferred to a new relationship.

In addition, the majority of those who stayed with the cancer patient reported that the cancer influenced the relationship. However, a negative influence was described more frequently. Depression and relationship satisfaction were found to be significant factors in the type of impact cancer had on the relationship (positive or negative). Higher levels of depression were associated with more negative perceptions, whereas a more satisfied relationship tended to be associated with more positive perceptions. Demographic variables such as age or gender did not appear to predict perceptions of cancer. It is possible that this perception may be due to a caregiving burden associated with partner strain. Although not all cancer patients are in need of caregivers, spousal caregivers are at higher risk for mental, physical and social morbidity due to their caregiving experience (Li and Loke, 2013). In particular, the burden of caregiving appears to have an impact on the psychological distress of the partner (Geng et al., 2018). Unfortunately, data on caregiving burden are not available in this study.

Those who separated showed higher depression scores compared with those who stayed together and were more likely to have had psychological treatment in the past. Those who had no history of psychological treatment, had lower anxiety levels, and lower partnership satisfaction were at increased risk for relationship dissolution. The odds ratio for anxiety and relationship satisfaction was above 1, at 1.22 for anxiety and 1.04 for relationship satisfaction. Specifically, psychological variables appeared to predict separation, but also perceptions of the impact of cancer on the relationship as positive and negative. In contrast, medical and/or sociodemographic factors do not seem to be relevant. This is consistent with other studies showing that anxiety rather than depression was most of a problem in long-term cancer survivors and spouses compared to healthy controls (Mitchell et al., 2013).

It is important to acknowledge that this study has some limitations. First, this study included partners of patients with different types of cancers, resulting in heterogeneity. This may be considered both an advantage and a disadvantage. Second, the assessment of the impact of cancer on the relationship may be subject to subjective bias because the assessment was retrospective. Participants may either overestimate or underestimate the impact of cancer on the relationship. Third, it was not possible to determine when the relationship ended after the cancer diagnosis. Thus, the direction of causality between relationship dissolution and cancer diagnose could not be determined. Although cancer diagnosis is not a direct causal factor, the study suggests that non-causal associations may exist and that these associations are important with regard to the vulnerability of divorced partners of cancer patients. Forth, no information was available on who initiated the separation. Fifth, a higher response rate might have captured more cases of relationship dissolution because participants who refused to complete the survey may have faced more serious problems than those who accepted it. Finally, cancer-related mortality may further contribute to the underestimation of relationship dissolution and the effects of cancer on relationship. Despite the limitations, strengths of the study include its focus on partners of cancer patients, who have not previously been the focus of studies of cancer and separation, and its extensive data, which also allow for more sophisticated analyses, such as predictors of separation and the impact of cancer on a relationship.

The results show that the partners of cancer patients also suffer long-term from the consequences of cancer. Assuming that a good relationship is a protective factor for the patients (Buja et al., 2018) and that a cancer diagnosis can be a burden for both – the patient and the partner (“we-disease”; Kayser et al., 2007), partners should therefore also be considered in care and the focus should be on the relationship as well as the psychological stability of the partners. Interventions aimed at improving psychological functioning and quality of life of cancer patients and their partners are necessary to reduce negative effects on the individuals and also the couple.

## Data Availability Statement

The raw data supporting the conclusions of this article will be made available by the authors, without undue reservation.

## Ethics Statement

The studies involving human participants were reviewed and approved by Hannover Medical School (number 3653-2017) and the University Hospital Düsseldorf (number 2017114500). The patients/participants provided their written informed consent to participate in this study.

## Author Contributions

TZ and AK conceptualized and designed the study. BN collected the data of the study. TZ, AK, and BN analyzed and interpreted the data. TZ and BN wrote the original draft of the manuscript. TZ, AK, and BN reviewed and edited the manuscript. All authors contributed to the article and approved the submitted version.

## Conflict of Interest

The authors declare that the research was conducted in the absence of any commercial or financial relationships that could be construed as a potential conflict of interest.
