# Supplementary material for: Cancer and Relationship Dissolution: Perspective of Partners of Cancer Patients
Source: Front Psychol. 2021 May 21;12:624902. doi: 10.3389/fpsyg.2021.624902 (PMC8177048; doi:10.3389/fpsyg.2021.624902)
Supplement: Supplementary file 1 [file Data_Sheet_1.docx]

Supplementary Material

# Supplementary Data

All questionnaires used in this study are listed in the publication.

To answer research question 2 (Impact of cancer on the relationship), the partners' assessment of the subjective impact of cancer on the relationship was used (Has cancer affected your relationship? If yes, positively or negatively?).

To answer research question 3 (factors influencing relationship dissolution), we analyzed sociodemographic factors of the partners such as age, gender, separation of own parents, children or medical factors (own disease, psychological treatment) and psychological factors such as depression, anxiety, distress, quality of life and relationship satisfaction. These factors were selected based on the existing literature on factors influencing separation, but these factors have not previously been analyzed in the context of cancer and separation.

**
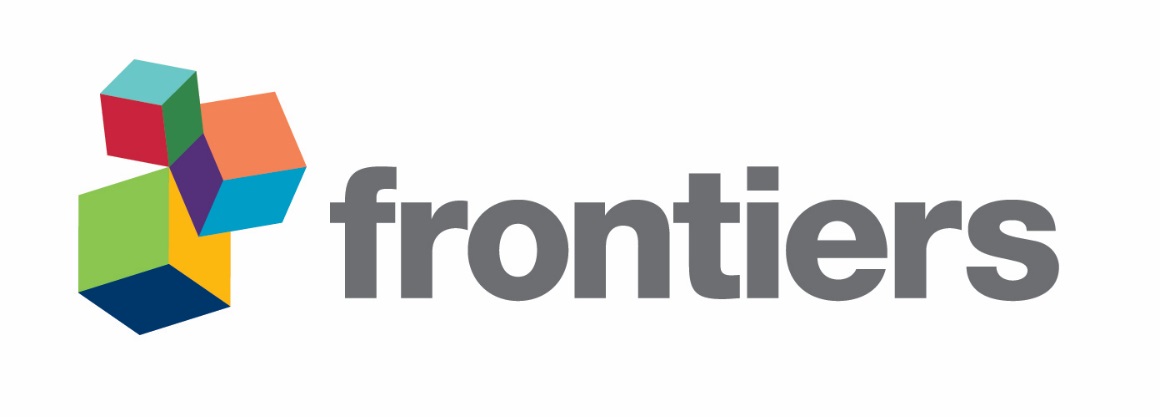
**
